# Processing renewable and waste-based feedstocks with fluid catalytic cracking: Impact on catalytic performance and considerations for improved catalyst design

**DOI:** 10.3389/fchem.2023.1067488

**Published:** 2023-01-19

**Authors:** Melissa Clough Mastry, Lucas Dorazio, James C. Fu, Juan Pedro Gómez, Sergio Sedano, Snehesh S. Ail, Marco J. Castaldi, Bilge Yilmaz

**Affiliations:** ^1^ BASF Corporation, Iselin, NJ, United States; ^2^ Consultant, Madrid, Spain; ^3^ Neoliquid Advanced Biofuels and Biochemicals, Guadalajara, Spain; ^4^ Chemical Engineering, City College of New York, New York, NY, United States

**Keywords:** fluid catalytic cracking, sustainability, renewable feedstocks, pyrolysis oil, biofuels, waste to energy, biomass upgrading, industrial waste

## Abstract

Refiners around the globe are either considering or are actively replacing a portion of their crude oil inputs originating from fossil sources with alternative sources, including recycled materials (plastics, urban waste, mixed solid waste) and renewable materials (bio-mass waste, vegetable oils). In this paper, we explore such replacement, specifically focusing on the fluid catalytic cracking (FCC) operation. Five pyrolysis oils, obtained from municipal solid waste (MSW) and biogenic material (olive stones/pits), were fully characterized and tested at 10% loading against a standard fluid catalytic cracking (FCC) vacuum gasoil (VGO) feed in a bench scale reactor using an industrially available fluid catalytic cracking catalyst based on ultrastable Y zeolite to simulate fluid catalytic cracking co-processing. Despite having unique feed properties, including high Conradson carbon (e.g., up to 19.41 wt%), water (e.g., up to 5.7 wt%), and contaminants (e.g., up to 227 ppm Cl) in some cases, the five pyrolysis oils gave similar yield patterns as vacuum gasoil. Gasoline was slightly (*ca*. 1 wt%) higher in all cases and LPG slightly (*ca*. 1 wt%) lower. Olefinicity in the LPG streams were unchanged, bottoms and light cycle oil (LCO) showed no significant changes, while dry gas was slightly (up to −0.2 wt%) lower. Coke selectivity was also unchanged (maximum −7.7 wt%, relatively), suggesting minimal to no heat balance concerns when co-processing in an industrial fluid catalytic cracking unit. The results demonstrate the applicability of municipal solid waste and biogenic originating pyrolysis oils into a refinery. A catalyst design concept is explored, based on higher rare Earth oxide exchange and/or utilization of ZSM-5 zeolite, that would further minimize the impacts of replacing fossil oils with pyrolysis oils, namely one that shifts the 1% higher gasoline into LPG.

## Introduction

The valorization of waste and renewable streams has been studied for many years (Tuck et al., 2012) (Sheldon, 2014) (Ragaert et al., 2017) (Kiran et al., 2014) (Dermeche et al., 2013) (Dahiya et al., 2018). However, more recently this concept is quickly gaining traction in industrial practices as more companies are trying to implement such strategies. In addition, from a consumer and regulatory perspective, the demand and push, respectively, for cleaner and more sustainable energy and material sources is increasing. Importantly, this includes the Renewable Energy Directive II (RED II), redefined in 2018, requiring 32% of renewable energy in the European Union by the year 2030 (European Commission, 2018), and more recently RED III, redefined again in 2022, requiring 45% renewable energy within the same time frame.

Renewable and recyclable waste can be broadly categorized into three categories: plastic waste, agricultural waste, and municipal solid waste (MSW), with the latter typically comprised of both plastic and agricultural wastes, among other materials. Dedicated plastic recycling can be done either mechanically or chemically; mechanical recycling is a proven viable option, while chemical recycling (Ragaert et al., 2017) [sometimes called advanced recycling and/or ChemCycling™ (Grauke et al., 2021)] offer other pathways, allowing for the decomposition of polymeric material into virgin molecules (i.e., ethylene and propylene). Chemical recycling is typically done thermochemically and can involve steam gasification (Saebea et al., 2020) or pyrolysis, with the ultimate goal to produce monomers and/or fuels (Lopez et al., 2017) (Wong et al., 2015). Steam gasification typically results in more chemical building blocks, whereas pyrolysis processes result in more fuel-range building blocks (Antelava et al., 2021).

Agricultural waste can be disposed of or re-used in a variety of ways. Common pathways include animal feed, composting, or landfilling, with pyrolysis upgrading becoming more prevalent (Lahijani et al., 2022). Many sources of agricultural waste and biomass have been studied *via* the pyrolysis pathway, including woodchips (Pinho et al., 2017) (Lutz et al., 2022) and food waste (Dahiya et al., 2018) (Kiran et al., 2014). In Europe, the waste from olive mills accounts for 9.6 million tons per year, with half of the European production originating in the southern part of Spain, Andalucía (Berbel and Posadillo, 2018). Most of this waste goes toward very low value-added processes, including the generation of heat and electricity, with only 5% going towards higher value-added processes including animal feed. In the median of the value addition scale is the generation of biofuels and chemicals (Berbel and Posadillo, 2018), suggesting that the transformation into biofuels and biochemicals is an attractive pathway.

At the intersection of plastic and agricultural waste is MSW, whose generation is nearly 2.0 billion metric tons and is expected to increase in the future, with some projecting MSW to comprise a large portion of the future biofuels market (Gelder et al., 2022). The investigation into MSW valorization is complex, given its diversity. Its composition varies with respect to region, waste management practices, and sorting complexity. Importantly, plastic in MSW on a global scale is approximately 12 percent by weight and, therefore, can be a significant source for chemical and fuel manufacture. The renewable portion, excluding yard and food waste, comprises about 34 wt% and represents a truly renewable path to fuels and chemicals. Various researchers and companies also investigate the use of more specific unique streams, including waste tires (Tian et al., 2022; Pyrum Innovations, 2022) and waste biological products (Shim et al., 2022). Laboratory experiments have been conducted on carefully prepared MSW-like materials (Sorum et al., 2001) and the pyrolysis of MSW has been studied in detail by many groups (Chen et al., 2014; Du et al., 2021). However, the post-processing of the resulting MSW-pyrolysis oil to generate valuable fuels and chemicals is less studied.

In this paper, we focus on the usage of pyrolysis oils from MSW and biomass waste in an FCC unit. The FCC is at the heart of the refinery and is one of the more flexible processes in the refinery, making it an ideal candidate for the processing of unique streams. By utilizing pilot scale laboratory testing, the impact of alternative feed sources on FCC product yields can be quantified by replacing a portion of the fossil-based feed. Given the flexibility of the FCC process and its ability to handle unique feedstocks and contaminants, it remains a very valuable tool when considering outlets for pyrolysis oil upgrading (Lappas et al., 2009; Fogassy et al., 2011). Previous literature exploring the co-processing of biogenic pyrolysis oils utilize lab-created pyrolysis oils (Magrini et al., 2022; Seiser et al., 2022). The novelty in this paper lies in the direct application to industry and the unique nature of the pyrolysis oils that are from an industrial scale operation and are already being sold into the open market. The utilization of industrially available pyrolysis oils and catalysts in this paper demonstrates the applicability of previous works to real life scenarios. The post-processing of the resulting pyrolysis oils is evaluated using an industrially available FCC catalyst. Further, the application of the pyrolysis oil is such that the co-processing level is industrially relevant, i.e., tested at a level that a typical refiner would also consider/utilize at scale. The work presented supports refiners’ goals to use existing equipment and infrastructure to answer the latest question around sustainability. These concepts (yield impacts, feed characterization, and contaminant handling) are of paramount importance to enable the refining industry’s partial or full replacement of fossil fuels.

## Experimental

Pyrolysis oils were obtained from the industrial plant of Neoliquid in Guadalajara (Spain) and from the industrial plant of Neoliquid/Preco in Toledo (Spain) and used without further pre-processing. Notably the BIO1 material is the oil product from pyrolysis, whereas the aqueous fraction (wood vinegar) obtained from pyrolysis was separated into a different product stream. Both plants operate using the pyrolysis technology of Neoliquid Biofuels and Advanced Biochemicals. The municipal solid waste (MSW) processed in these plants has previously been processed to remove glass, metal, and minerals. The feed samples spent 18 days in transit (in ambient and cold temperatures during ground and air transport) before being analyzed. All were dark and viscous liquids at room temperature. Upon arrival at the testing lab, they were stored in a freezer (0°C). In addition to the five pyrolysis oils, a standard vacuum gasoil (VGO) feed was also used. The oils were used as-received, without the use of stabilizing agents.

Six oils were included in this study, with descriptions listed below:

Standard: VGO representative of an FCC feed used in a typical US gulf coast FCC unit.

MSW1: Pyrolysis oil from MSW, containing 70%–90% mixed plastics and 10%–30% paper, cardboard, biomass, and textile waste.

MSW2: Heavy liquid fraction from the MSW1 pyrolysis process.

MSW3: Pyrolysis oil from MSW, containing 70%–90% mixed plastics (with a high fraction of polyolefins) and 10%–30% paper, cardboard, biomass, and textile waste.

BIO1: Pyrolysis oil from biomass waste (olive stones/pits).

MIX1: A 10% BIO1 and 90% MSW1.

The catalyst used in the cracking evaluations was a porous zeolite-based *in-situ* catalyst (McLean, 2003) containing a high ultrastable Y (USY) content designed for resid applications. The properties for fresh catalyst are found in Table 1. The catalyst was deactivated under hydrothermal conditions (100% steam, 788 C, 24 h) and this deactivated catalyst, properties for which are found in Table 2, was used for all catalytic evaluations.

**TABLE 1:** Fresh catalyst properties.

Parameter	Fresh catalyst property
Total surface area, m^2^/g	311
Zeolite surface area, m^2^/g	225
Matrix surface area, m^2^/g	86
Rare earth oxide, wt%	2.2
SiO_2_, wt%	50.2
Al_2_O_3_, wt%	44.6
Na2O, wt%	0.22
UCS, Å	24.59
APS, µ	80

**TABLE 2:** Steam deactivated catalyst properties.

Parameter	Deactivated catalyst property
Total surface area, m^2^/g	133
Zeolite surface area, m^2^/g	88
Matrix surface area, m^2^/g	45
UCS, Å	24.29

Elemental analysis of the catalysts was conducted by X-ray Florescence (XRF) using a Philips PW2400 spectrometer, with fused pellet specimens; data were accurate within 1% relative abundance. BET (TSA) surface area was determined using N_2_ adsorption data acquired using a Micromeretics Galaxy 3,060 sorptometer. T-plot external area (MSA) was calculated from the same N_2_ isotherms. Results have a precision of about ±2% relative.

Unit cell size was determined following ASTM D3942. X-Ray diffraction data were collected using a PANalytical MPD X’Pert Pro diffractometer, with Cu radiation and Si was used as an internal standard. The unit cell size uncertainty of our estimate ranges from 0.004 to 0.01 Å.

Catalytic measurements were made using an Advanced Catalytic Evaluation (ACE™) fluid bed reactor (Kayser, 1997). The ACE was operated using a steam-deactivated catalyst, at a temperature of 529°C, 60 s injection time, 1.125 inch injector height, and using a constant time on stream protocol to vary catalyst to oil ratios of 4, 5, 6, and 7. Pyrolysis oils were tested at 10% loading with the balance of 90% as a standard FCC feed. This approach is being considered by early adopters from a refinery perspective, with many opting to co-process alternative (e.g., pyrolysis) oils at 1%–10% loading. Gasoline product was defined as C_5_ to 232°C, diesel as 232°C–360°C, and bottoms as 360 °C and higher. API gravity, refractive index, and viscosity were measured at 16, 25, and 99°C, respectively. PIONA were not collected on the syncrude materials.

High temperature simulated distillation (HT SIMDIS ASTM 7500 method) of oil samples were conducted on an Agilent 7890 B GC equipped with a SIMDIS capillary column capable of reaching up to 430°C. This method starts at −20°C using cryogenic nitrogen and then ramps up to 430°C. Helium was used as carrier gas. Before oil samples were introduced to the GC, they were first diluted with carbon disulfide (CS_2_) at 1:50 ratio (0.02 g sample with 1 g CS_2_). The HT SIMDIS ASTM 7500 method was used to determine boiling point distribution for samples that could not elute completely by ASTM 2887 method and can determine boiling points from n-C5 to n-C120 distribution.

Water content in the oil samples were measured by a Karl Fischer titrator (Mettler Toledo V30S). A sample is added to dry methanol (no water present) and the mixture is titrated with a titrant (Composite 5) until it reaches the end point which is determined using bi-voltametric indication, i.e., the potential at the polarized double-platinum-pin electrode falls below a certain value (e.g., 100 mV). The water content of the sample is calculated from the amount of titrant used.

CHN (carbon, hydrogen and nitrogen) was measured by a LECO CHN628 analyzer. It is a combustion elemental carbon, hydrogen and nitrogen instrument that uses pure oxygen to completely combust a sample. Helium carrier gas sweeps the combustion gas to separate IR cells for detection of CO_2_ and H_2_O. A TCD (thermal conductivity detector) is used to detect nitrogen. Oxygen is calculated from balancing to 100. Other elements in oils were determined by ICP (inductively coupled plasma) using a method similar to ASTM D7691. The ICP instrument uses two-point calibration (blank and 1 ppm) and sample preparation involves kerosene as the diluent.

In addition to the physical and chemical analyses, a more detailed analysis of the hydrocarbons comprising each feedstock oil was performed using an Agilent 7890B gas chromatograph equipped with an Agilent 5977A mass spectrometer. For these measurements, the pyrolysis oil was dissolved in cyclohexane solvent at a ratio of 1:100 and the hydrocarbon spectra were characterized using a slightly polar SHRX5LB column.

For catalytic testing, the pyrolysis oils were mixed with standard feed at 10/90 weight ratio without the use of emulsifiers. Upon mixing, the two oils were miscible with no phase separation noted, distinctly different from other accounts of immiscibility of biogenic oils and standard petroleum-based oils (Seiser et al., 2022). The homogeneous feeds were then injected into the catalytic reactor through a singular injection line.

## Results and discussion

First, we examine catalyst and oil characterizations. For the catalyst, because the continuous operation of FCC units results in an aged distribution of particles, we must examine both fresh and deactivated catalysts. Fresh and steam deactivated catalysts tested are noted below.

Table 3 below describes the full (neat) feed analyses. The API gravities of the MSW and MIX1 pyrolysis oils are comparable to the standard VGO feed. The BIO1 pyrolysis oil gave a negative API value suggesting a heavy feed, confirmed by distillation. In terms of Conradson carbon, BIO1 oil gives values that are much higher than those currently being processed in FCC units globally while the MSW1-3 and MIX1 oils falling in a more traditional range (BASF benchmarking database). However, when considering the later evaluation at the 10% co-processing scenario, the resulting weighted average even with the BIO1 oil is well within typical FCC feeds. Not surprisingly, the BIO1 feed gives considerable water content, typical of bio-based feedstocks (Magrini et al., 2022), while the MSW and MIX1 feeds have more typical water content. The very high viscous nature of the BIO1 sample precluded its viscosity from being accurately measured. Similarly, the pour points for MSW3 and BIO1 were not measured because portions of these two samples remain as liquid at lowest temperature (−6 C) achievable for the instrument.

**TABLE 3:** Properties of feeds used in this study.

	Standard	MSW1	MSW2	MSW3	BIO1	MIX1
Physical properties						
API (specific) gravity	23.79 (0.91)	33.71 (0.86)	24.86 (0.91)	31.16 (0.87)	−5.35 (1.12)	31.58 (0.87)
Conradson carbon (wt%)	0.43	1.09	3.85	0.44	19.41	1.65
Pour point (°C)	38	4	38	NM^b^	NM^b^	10
Aniline point (°C)	77	>93	>93	>93	>93	>93
Sulfur (wt%)	0.72	ND^a^	ND^a^	ND^a^	0.02	ND^a^
Refractive index	1.51	1.48	1.51	1.50	1.56	1.49
Viscosity (cSt)	7.43	1.14	5.64	1.37	NM^b^	3.53
Flash point (°C)	>149	38	>149	45	>149	38
Water (wt%)	0.07	0.01	0.17	0.22	5.72	0.08
Elemental analyses						
C (wt%)	86.4	84.5	85.7	84	67.9	84.6
H (wt%)	12.5	11.2	11.5	10.4	7.3	10.8
N (wt%)	0.11	0.08	0.19	0.08	0.32	0.13
O (wt%)	0.38	3.55	2.05	4.85	24.04	3.85
Br (ppm)	<1.5	16.3	22.3	7.1	<0.5	13.6
Ca (ppm)	<0.1	0.4	0.5	45.1	4.9	0.4
Cl (ppm)	<0.0001	198	227	205	17	194
Cu (ppm)	<0.1	0.2	0.4	0.8	1.2	0.2
Fe (ppm)	<0.1	9	8	43	61	2
K (ppm)	21	9	7	15	51	9
Mg (ppm)	<0.1	<0.1	0.1	7.2	1.0	<0.1
Na (ppm)	0.3	3	3	23	26	3
Ni (ppm)	0.3	0.2	0.9	0.4	0.6	0.2
P (ppm)	<0.1	11.1	35.1	5.5	6.2	11.2
Si (ppm)	0	90.8	36.4	60.2	4.7	93.4
V (ppm)	0.2	<0.1	<0.1	<0.1	<0.1	<0.1
GC Distillation (°C)						
Initial BP	251	61	147	43	96	99
5%	294	114	227	115	141	118
10%	338	138	272	139	187	137
20%	365	150	311	147	211	153
30%	388	177	343	148	236	193
40%	409	217	376	183	257	232
50%	428	253	410	238	288	268
60%	449	289	442	303	333	299
70%	472	329	481	369	363	341
75%	484	361	501	403	389	370
80%	496	393	522	437	416	399
85%	511	437	549	476	446	438
90%	526	482	576	516	477	476
95%	571	545	614	566	549	557
Final BP	616	643	665	642	622	637

^a^ ND, not detected.

^b^ NM, not measured.

The pyrolysis oils bring halogen contaminants, particularly Cl which range between 190 and 230 ppm for the MSW-originating oils (MSW1-3 and MIX1). The purely biogenic feed (BIO1) brings lower Cl at 17.3 ppm. The effects of Cl in FCC have been previously examined by our group (Senter et al., 2021). We do not expect to observe any effects in lab-scale testing considering the lack of nickel contaminant. In a refinery setting, care must be taken in case of nickel reactivation and chloride deposits downstream of the FCC unit.

**FIGURE 1:**
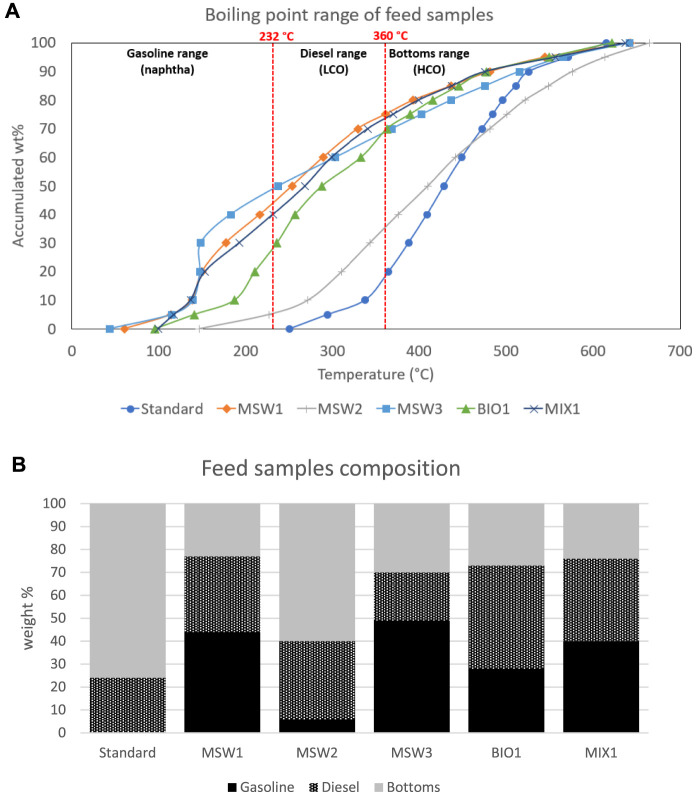
Standard and pyrolysis oil feed distillation curves **(A)**, top and component breakdown **(B)**, bottom.

The feed distillation is depicted graphically to provide a visual comparison. In Figure 1A, the pyrolysis oil distillations are plotted along with the standard feed. In most cases, the pyrolysis oils are lighter than the standard feed, with the exception of the MSW2 sample. This is not surprising considering that this is the heavy fraction from the pyrolysis process corresponding to MSW1. The feeds were further analyzed by the composition breakdown into gasoline, diesel, and bottoms using 232°C and 360°C cut points between the naphtha/LCO and LCO/HCO products, respectively, and are also depicted below in Figure 1B. The standard FCC feed contains a majority amount of bottoms product. Refiners typically process the standard feeds upstream to remove as much of the upgraded products as possible. However, in the case of the pyrolysis oils, we observe significant amounts, sometimes up to 75%, of the lighter gasoline and diesel products.

**FIGURE 2:**
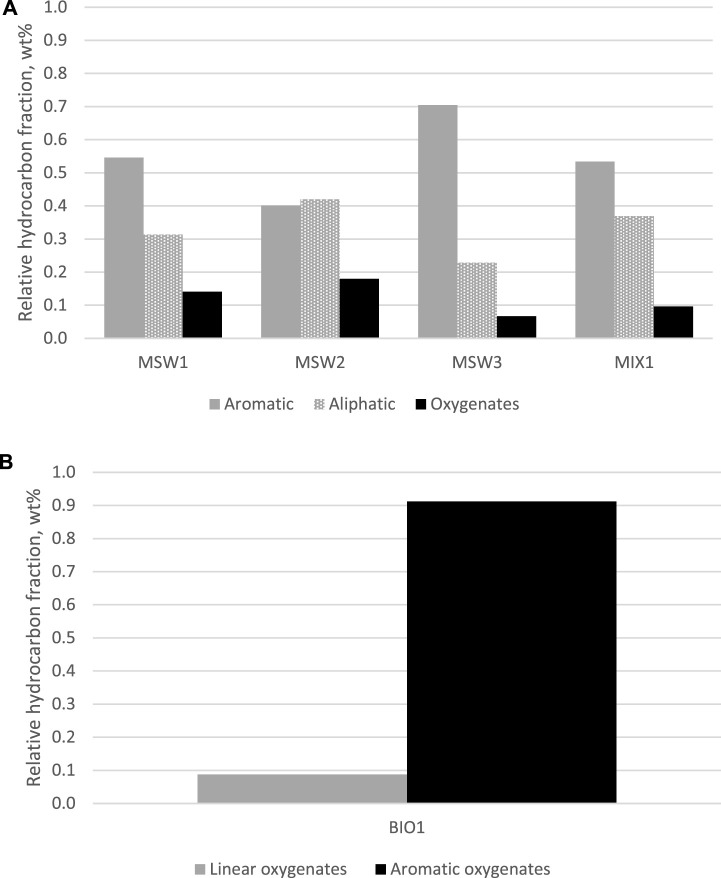
Dominant compounds found in pyrolysis oils; relative aromatic, aliphatic, and oxygenate hydrocarbon distribution in MSW-derived pyrolysis oils **(A)**, top and relative linear and aromatic oxygenate hydrocarbon distribution in biomass-derived pyrolysis oil **(B)**, bottom.

In addition to the bulk properties, GC-MS analyses were also conducted on the 5 pyrolysis oils to characterize their molecular makeup. The hydrocarbon spectra and the list of dominant compounds are provided in the Supplementary data (Supplementary Figures S1–S5; Supplementary Table S3). For the purposes of this analysis, MIX1 is characterized as an MSW-derived pyrolysis oil given the larger content of MSW1 *versus* BIO1. The MSW-derived pyrolysis oils contain aromatic, aliphatic, and oxygenated hydrocarbons, whereas the BIO1 sample is characterized by large fractions of cyclic-oxygenated hydrocarbons and small fractions of linear-oxygenated hydrocarbons. The distribution of these classes of hydrocarbons in the five pyrolysis oil samples are shown in Figures 2A,B.

Upon further analysis, not surprisingly, the biogenic oil BIO1 is distinctly different from the MSW oils. BIO1 was derived from olive pits/stones, which is a typical example of lignocellulose waste. In general, all lignocellulose waste will be comprised of lignin, hemicellulose, and cellulose. During pyrolysis, these highly oxygenated structural components of the biomass will undergo deconstruction and are expected to yield a highly diverse mixture of oxygenated hydrocarbons (Huber et al., 2006; Liu et al., 2014). Lignin will primarily yield phenols and guaiacols. Cellulose and hemicellulose will yield a diverse mixture of smaller carbonyl compounds and sugar derived compounds such as furfural and furan/pyran ring structures. Our analysis of BIO1, which is illustrated in the Supplementary data, is consistent with these expectations and shows the presence of phenolic molecules (phenol and substituted phenols) and other oxygenated hydrocarbons including ketones, fatty acids, and caffeine. There is a notable absence of light water-soluble compounds, i.e., those found after pyrolysis of cellulosic materials; this is likely explained by the separation of BIO1 oil from the aqueous fraction as explained in the experimental section. The composition of the MSW derived oils is quite different. Consistent with their oxygen content, there was a limited presence of oxygenates that included molecules such as large linear alcohols and esters. The bulk of the oil was comprised of aromatic and aliphatic hydrocarbons. Consistent with the oil Conradson carbon and specific gravity reported in Table 3, MSW 2 was comprised of larger aliphatic hydrocarbons and multiring aromatic structures.

Next, we examine the catalytic cracking evaluations, all of which were done using the deactivated catalyst as described in the experimental section. In the refining industry, it is customary to discuss catalyst activity in terms of the conversion to gasoline and lighter products, which is defined as 100—LCO—bottoms. At a constant catalyst to oil of 7, the conversion of all oils were slightly higher than the standard feed, which gave 75.8 wt% conversion. MSW1, MSW2, MSW3, BIO1, and MIX1 gave conversion levels of 76.2, 75.9, 76.1, 76.3, and 76.2 wt%, respectively. This is likely due to already converted material (i.e., naphtha range molecules) entering as feed in all cases. To examine the effects on product selectivities, the cracking evaluations are examined at iso conversion (75 wt%), obtained by regression of raw data. This conversion value was chosen since it is the median value of all tests and does not require extrapolation. Compared to the standard feed, all pyrolysis oils gave an increase in gasoline yield and a decrease in LPG yield. It is likely this shift is the result of higher aromatic content in the naphtha fraction of the pyrolysis oils relative to the standard gasoil. During catalytic cracking, these aromatics will be relatively inert and ultimately collected within the gasoline product fraction. Coke selectivity is the same or lower for all pyrolysis oils compared to the standard oil, suggesting that major heat balance issues in an FCC unit would not arise or would be minimal with the co-processing of these oils. In the case of minimal heat balance selectivity (e.g., with MSW3 at −7.7% on a relative basis), delta coke in the unit could be increased. The small shift in LCO and bottoms yield is likely the result of the higher natural fractions of naphtha and LCO sized molecules contained in the pyrolysis oil feedstocks. Unlike other studies of biogenic pyrolysis oil coprocessing in an FCC (Pinho et al., 2017), the processing of the biogenic material (BIO1 coming from olive stones/pits) did not result in lower liquid yields.

The olefinicity, which is the ratio of cracking (olefin producing) and hydrogen-transfer reactions (olefin consuming) is an important metric in FCC analysis. The LPG selectivities (LPG olefinicity, C3 olefinicity, and C4 olefinicity) are not affected in any of the pyrolysis oil co-processing scenarios. Dry gases are either similar (BIO1) or lower than the standard feed, suggesting that dry gas handling during a co-processing event in an FCC unit would not be a concern. Yields of products of deoxygenation, i.e., CO, CO_2_, and H_2_O, are also listed in Table 4. The oxygen content of the standard gasoil and yields of CO, CO_2_, and H_2_O should approach zero. Yields of these products reported in Table 4 are the result of either measurement error and/or atmospheric water inadvertently introduced into the recovered syncrude liquid as it is prepared for external analysis. The yields of CO, CO_2_, and H_2_O for the standard feed should be considered a zero baseline to compare the other feedstocks against. For all cases, the yields of CO and CO_2_ are similar to that of the standard gasoil. This suggests that at least for the case where the co-processing is limited to 10%, deoxygenation through decarbonylation (yielding CO) or decarboxylation (yielding CO_2_) is not significant enough to be detected in product yields. Yield of water is consistent with the oxygen content of the feedstock where the water yield from the lower oxygen containing feedstocks were similar to the standard gasoil. Only for the case of BIO1 was the water yield higher, suggesting the preferred pathway for deoxygenation was likely dehydration. In terms of FCC co-processing, the production of water would translate to more wastewater treatment and processing. In terms of FCC chemistry, production of water would consume hydrogen and potentially lead to higher coke yields, although this was not observed for the 10% co-processing of BIO1. Not explored in this paper are potential impacts on equipment corrosion and fouling in an industrial FCC unit.

**TABLE 4:** Catalytic cracking evaluation of all feeds at constant conversion (75 wt%).

	Standard	MSW1	MSW2	MSW3	BIO1	MIX1
Process variables						
Catalyst to oil ratio, wt/wt	6.65	6.38	6.50	6.30	6.35	6.41
Hydrocarbon yields						
H_2,_ wt%	0.08	0.08	0.08	0.08	0.07	0.08
Methane, wt%	0.88	0.82	0.84	0.79	0.86	0.81
Ethane, wt%	0.59	0.55	0.59	0.55	0.58	0.57
Ethylene, wt%	0.81	0.76	0.78	0.75	0.84	0.77
Propane, wt%	1.07	0.99	1.01	0.96	1.03	0.97
Propylene, wt%	5.83	5.55	5.72	5.49	5.67	5.56
*n*-Butane, wt%	0.89	0.82	0.84	0.81	0.86	0.81
*i*-Butane, wt%	4.11	3.84	3.88	3.76	3.95	3.75
*n*-Butenes, wt%	5.32	5.11	5.33	5.11	5.20	5.20
*i*-Butylene, wt%	2.26	2.22	2.34	2.24	2.24	2.30
Gasoline, wt%	50.42	51.63	50.86	51.94	50.96	51.59
LCO, wt%	15.18	15.23	15.36	15.22	15.29	15.34
Bottoms, wt%	9.82	9.77	9.64	9.78	9.71	9.66
Coke, wt%	2.73	2.64	2.75	2.52	2.74	2.59
Calculated values						
Total valuable liquids^a^, wt%	85.08	85.39	85.33	85.54	85.20	85.52
Total dry gas, wt%	2.36	2.20	2.28	2.16	2.35	2.23
Total LPG, wt%	19.49	18.53	19.11	18.38	18.95	18.59
LPG olefinicity, wt/wt	0.69	0.70	0.70	0.70	0.69	0.70
Total C4=, wt%	7.58	7.33	7.67	7.36	7.44	7.50
C3 olefinicity, wt/wt	0.84	0.85	0.85	0.85	0.85	0.85
C4 olefinicity, wt/wt	0.60	0.61	0.62	0.62	0.61	0.62
Non-hydrocarbon yields						
CO, wt%	0.05	0.04	0.06	0.05	0.09	0.06
CO_2_, wt%	0.26	0.31	0.28	0.24	0.26	0.25
H_2_O, wt%	0.82	0.75	0.81	0.42	1.15	0.31

^a^ Total valuable liquids defined as LPG + gasoline + LCO.

**FIGURE 3:**
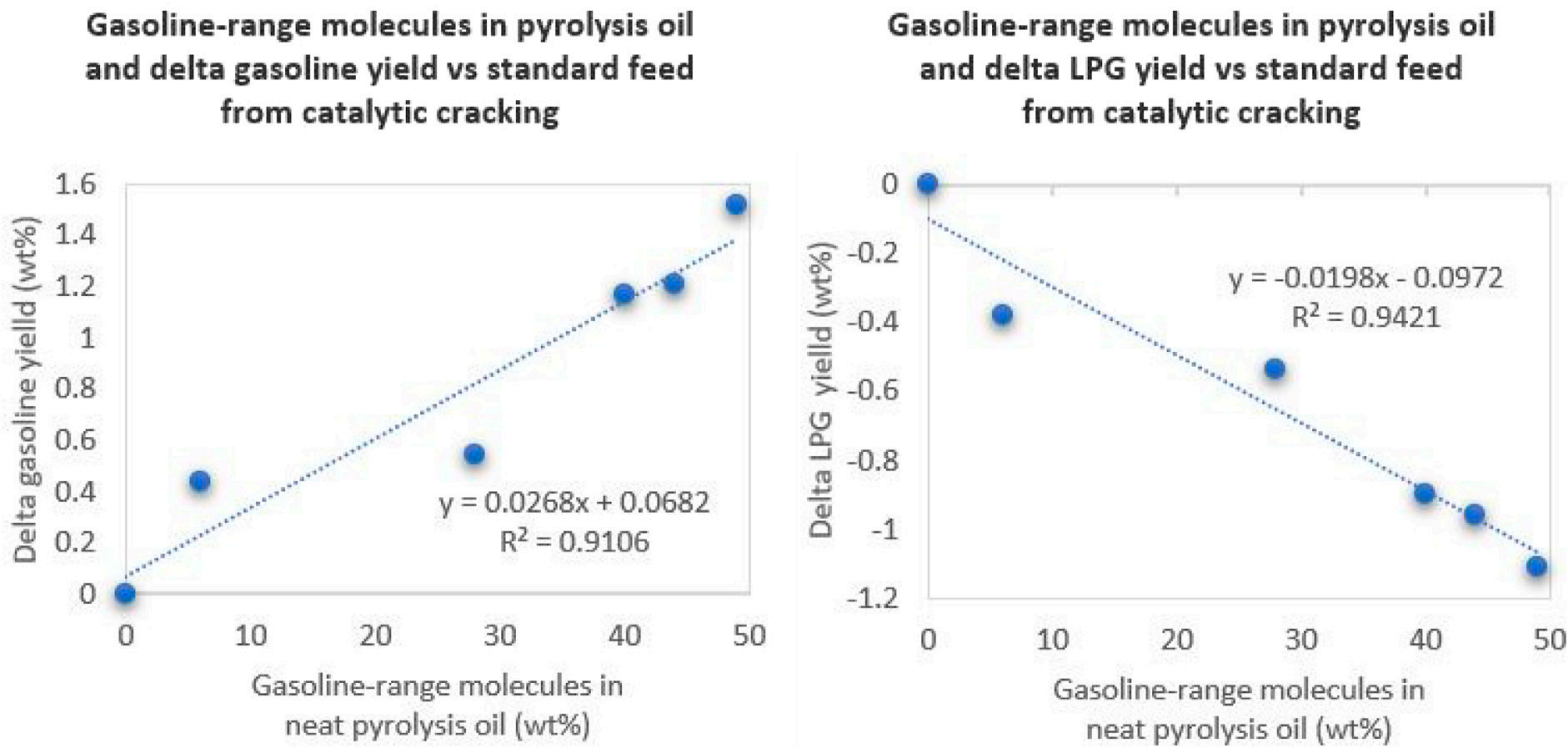
Correlations between gasoline-range molecules in the pyrolysis oils and resulting gasoline (left) and LPG (right) yield deltas from the catalytic cracking evaluations.

There is a correlation (*R* ^2^ = 0.91) between the amount of gasoline-range molecules in the neat pyrolysis oil (presented in Figure 1 above) and the delta gasoline yield *versus* standard feed (presented in Table 4 above). The pyrolysis oil coming with the highest amount of gasoline-range molecules (MSW3, 49%) also gives the highest delta gasoline yield (+1.52 wt%) *versus* the standard feed in the catalytic cracking evaluation. There also is a similarly good correlation (*R* ^2^ = 0.94) between the amount of gasoline-range molecules in the neat pyrolysis oil and the delta LPG yield *versus* standard feed, in this case showing a negative trend. Both correlations are shown below in Figure 3. Other possible correlations between diesel content in the pyrolysis oils, bottoms content in the pyrolysis oils, LPG + gasoline delta yields, and bottoms delta yields, were determined to not have relevant (*R* ^2^ > 0.90) correlations, suggesting that gasoline-range molecules in the pyrolysis oil is the most relevant bulk property in terms of resulting catalytic cracking differences. We theorize that the gasoline made from the pyrolysis oils are distinctly different from the gasoline made from the VGO oil. The data suggest that the naphtha fraction from the pyrolysis oil does not undergo significant further cracking into LPG. This theory is supported by the above observations of high aromatics content of the pyrolysis oils. Since FCC units do not crack aromatic compounds, the aromatics remain in the gasoline product cut as a high-octane naphtha molecule. The expected difference in gasoline composition is important for a refiner to understand, especially if further cracking by additional zeolites, i.e. ZSM-5, are used. Since ZSM-5 works mainly on linear and near linear naphtha molecules, the higher presence of aromatic content within the naphtha range will result in lower secondary cracking.

For refiners and researchers who prefer to examine the data at constant catalyst-to-oil ratio and at constant coke, we offer the full cracking evaluation data in Supplementary Tables S1 and S2 as part of the supporting information. As expected in the constant coke cases, the small coke selectivity differences are translated in different conversion levels, with all pyrolysis oils being higher than the standard oil.

We further evaluated various pragmatic cases in terms of pyrolysis oil crackability, an exercise that a refining organization might likely perform themselves during the planning stages of such an undertaking. In this analysis, we examined two cases.

- Scenario 1: in which we assumed pure carry-over of the pyrolysis oil components and no further conversion
- Scenario 2: in which we assumed standard conversion of the unconverted products in the pyrolysis oils

For all scenarios, the catalyst to oil of 7 data were used, in which the standard oil delivered a conversion of 75.8 wt%. As an illustration of scenario 1, the MSW1 contains 56 wt% unconverted products (i.e., diesel and bottoms). Applying a 75.8 wt% conversion to that figure, the remaining diesel and bottoms content of a pure MSW1 would be 13.6 wt% [i.e., 56—(56 * 0.758)]. The theoretical conversion of the co-processed MSW1 is then a weighted average of the VGO conversion (75.8 wt%) and of the pyrolysis conversion (100—13.6 wt%), giving a value of 76.9 wt%. These calculations were repeated for the other pyrolysis oils.

To illustrate scenario 2, the natural conversion level (i.e., naphtha content) of the pyrolysis oil was considered. For MSW1, this natural conversion level is 44 wt%. The theoretical conversion therefore is a weighted average of the VGO conversion (75.8 wt%) and the natural conversion level of the pyrolysis oil (44 wt%), giving a value conversion of 76.9 wt%.

**FIGURE 4:**
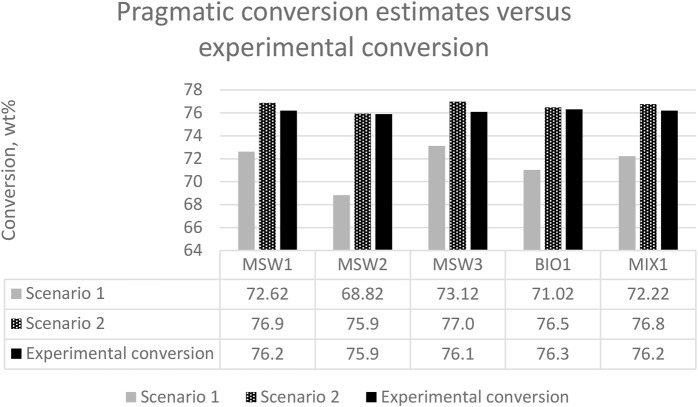
Pragmatic conversion estimates using two scenarios compared to observed experimental conversion.

The two scenarios were then compared to the experimental (observed) conversion to give further insight into the pyrolysis oil reactivity, with the key data summarized in Figure 4. The full data tables explaining these calculations and estimates can be found in Supplementary data (Supplementary Table S3).

We found that the experimental conversion was always higher than scenario 1 and lower than scenario 2. The higher observed conversions *versus* scenario 1 conversions illustrate that the pyrolysis oils are indeed undergoing cracking reactions, rather than a pure carry-over of existing converted and heavy products. The lower observed conversion *versus* scenario 2 conversions illustrates that the heavy fractions from the pyrolysis oils are more difficult to convert than the standard VGO, which is supported by the high aromaticity of the pyrolysis oils.

## Conclusions

Five pyrolysis oils originating from MSW and/or biogenic materials (olive stones/pits) were compared against a standard VGO material for FCC co-processing applications. The pyrolysis oils were mixed with standard oil at 10/90 ratio with no issues with miscibility. The catalytic results show very favorable outcomes for the co-processing of MSW and bio- originating pyrolysis oils with minimal impacts. An increase in valuable liquids was observed, likely due to the high qualities/properties of BIO1 compared to other biomass pyrolysis liquids. In fact, all pyrolysis oils gave higher total valuable liquid yields. This catalytic evaluation suggests that the co-processing of the 5 pyrolysis oils studied could be rather straight forward for some refineries and FCC units with respect to yield selectivities. Some impacts on product qualities are expected, namely a more aromatic naphtha product. Further considerations for a refinery would be the handling of any unique contaminants that cannot be assessed in small scale laboratory settings, such as the effect of chlorides and possible corrosion or fouling in an FCC unit. The pragmatic conversions estimate analysis suggests that the pyrolysis oils are indeed undergoing further cracking of the heavy (diesel and bottoms) fractions and not simply exhibiting a carryover effect. The experimental conversion was lower than the scenario 2 case, suggesting that the heavy molecules in pyrolysis oils are slightly more difficult to crack than the standard VGO.

Given the impact on product selectivities have been mainly towards gasoline and LPG, considerations for catalyst design are feasible. In the case where the refinery desires similar yields, i.e., ca. 1 wt% lower gasoline and ca. 1 wt% higher LPG to better mimic the standard feed case, a simple catalyst reformulation and/or usage of an LPG olefins additive would be beneficial, however taking into consideration the possibility of lower light olefins in the naphtha product fraction for further cracking by ZSM-5. Importantly, this study describes the “drop-in scenario”, i.e., co-processing pyrolysis oils without pre-treatment. In some cases, a refinery might elect to pre-treat the pyrolysis oil (through hydrotreating or another method) to lower contaminants going into the FCC unit. In another scenario, a refiner might elect to first process the pyrolysis oil using separation methods (i.e., distillation) to first remove the valuable fractions (i.e., gasoline and diesel) and feed the remaining heavy portions into the FCC unit.

## Data Availability

The original contributions presented in the study are included in the article/Supplementary Material, further inquiries can be directed to the corresponding author.
