# Supplementary material for: Processing renewable and waste-based feedstocks with fluid catalytic cracking: Impact on catalytic performance and considerations for improved catalyst design
Source: Front Chem. 2023 Jan 19;11:1067488. doi: 10.3389/fchem.2023.1067488 (PMC9893771; doi:10.3389/fchem.2023.1067488)
Supplement: Supplementary file 1 [file DataSheet1.docx]

**Supporting Information**

**Processing renewable and waste-based feedstocks with Fluid Catalytic Cracking: Impact on catalytic performance and considerations for improved catalyst design**

Melissa Clough Mastry^1^, Lucas Dorazio^1^, James C. Fu^1^, Juan Pedro Gómez^2^, Sergio Sedano^3^, Snehesh S. Ail^4^, Marco J. Castaldi^4^, Bilge Yilmaz^1, *^

^1^ BASF Corporation, 25 Middlesex/Essex Turnpike, Iselin NJ 08830 USA

^2^ Freelance Consultant in Oil Refining/Biofuels Technology, Avenida Juan Carlos I, nº 114, 2º2, 28916 Leganes, Madrid Spain

^3^ Neoliquid Advanced Biofuels and Biochemicals, Calle Mago de Oz, nº 7, 1ºC, 19005 Guadalajara Spain

^4^ Chemical Engineering, City College of New York, 140^th^ Street, New York NY 10031 USA

*Corresponding author: [bilge.yilmaz@basf.com](mailto:bilge.yilmaz@basf.com)


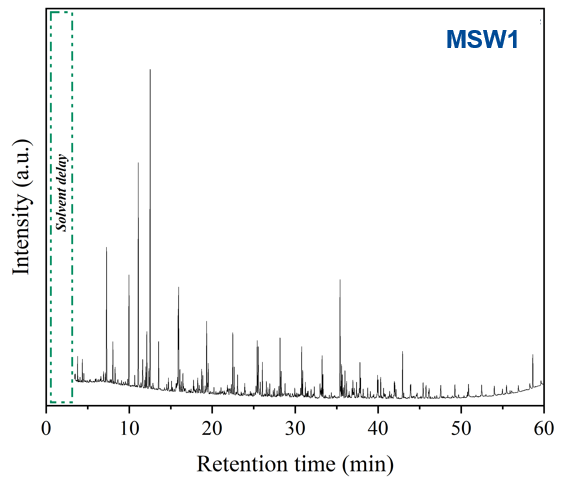


Figure S1. GS-MS spectrum of MSW1.


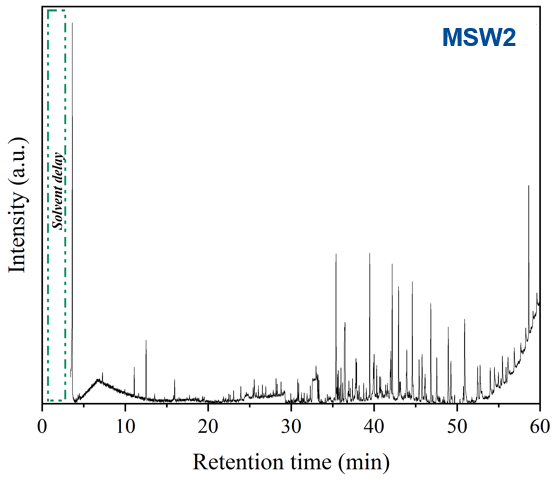


Figure S2. GS-MS spectrum of MSW2.


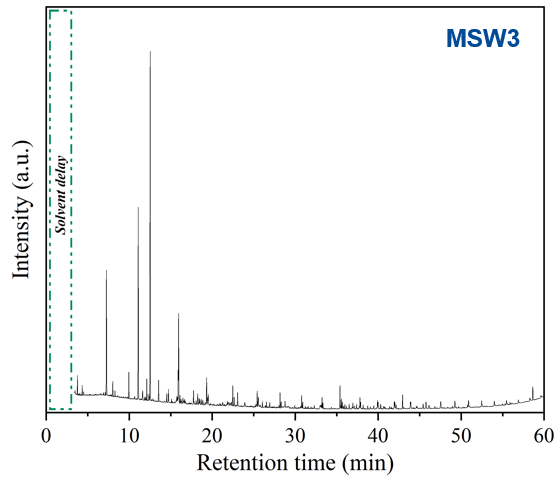


Figure S3. GS-MS spectrum of MSW3.


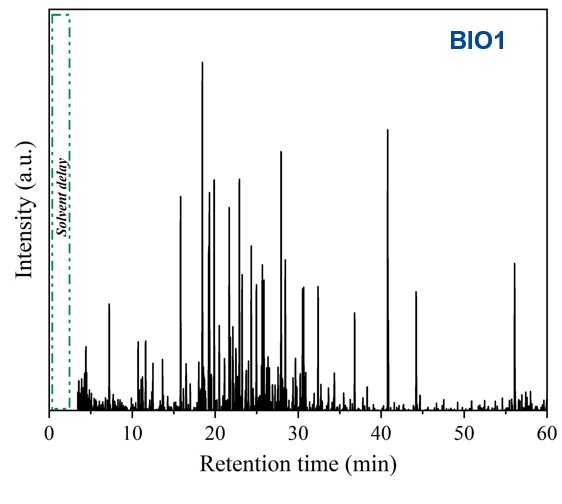


Figure S4. GS-MS spectrum of BIO1.


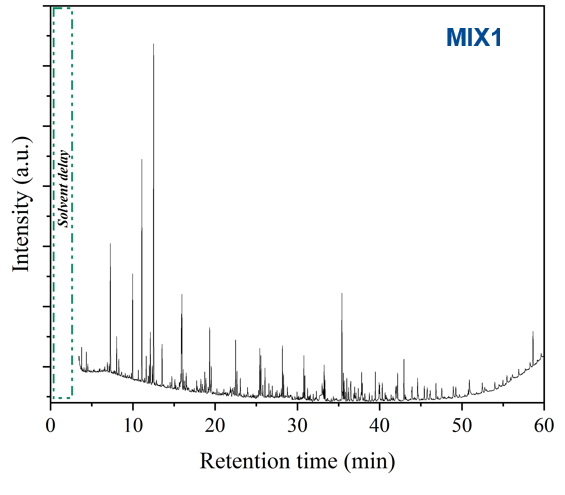


Figure S5. GS-MS spectrum of MIX1.

Table S1. Catalytic cracking evaluation of all feeds at constant catalyst to oil ratio (7 wt/wt)

|  | Standard | MSW1 | MSW2 | MSW3 | BIO1 | MIX1 |
| --- | --- | --- | --- | --- | --- | --- |
| Process variables | | | | | | |
| Catalyst to oil ratio, wt/wt | 7.0 | 7.0 | 7.0 | 7.0 | 7.0 | 7.0 |
| Conversion, wt% | 75.8 | 76.2 | 75.9 | 76.1 | 76.3 | 76.1 |
| Hydrocarbon yields | | | | | | |
| H_2_, wt% | 0.08 | 0.08 | 0.08 | 0.08 | 0.07 | 0.08 |
| Methane, wt% | 0.91 | 0.88 | 0.88 | 0.85 | 0.93 | 0.85 |
| Ethane, wt% | 0.60 | 0.58 | 0.60 | 0.58 | 0.60 | 0.59 |
| Ethylene, wt% | 0.83 | 0.81 | 0.81 | 0.80 | 0.90 | 0.81 |
| Propane, wt% | 1.12 | 1.07 | 1.06 | 1.04 | 1.12 | 1.03 |
| Propylene, wt% | 5.94 | 5.81 | 5.86 | 5.72 | 5.90 | 5.74 |
| *n*-Butane, wt% | 0.93 | 0.89 | 0.89 | 0.86 | 0.92 | 0.86 |
| *i*-Butane, wt% | 4.27 | 4.11 | 4.08 | 4.00 | 4.22 | 3.95 |
| *n*-Butenes, wt% | 5.40 | 5.26 | 5.40 | 5.24 | 5.29 | 5.30 |
| *i*-Butylene, wt% | 2.26 | 2.25 | 2.33 | 2.26 | 2.23 | 2.31 |
| Gasoline, wt% | 50.59 | 51.70 | 51.03 | 52.02 | 51.16 | 51.80 |
| LCO, wt% | 14.83 | 14.73 | 15.00 | 14.79 | 14.77 | 14.89 |
| Bottoms, wt% | 9.39 | 9.04 | 9.11 | 9.07 | 8.93 | 9.06 |
| Coke, wt% | 2.84 | 2.79 | 2.89 | 2.68 | 2.95 | 2.73 |
| Calculated values |  |  |  |  |  |  |
| Total valuable liquids^1^, wt% | 85.35 | 85.81 | 85.64 | 85.94 | 85.62 | 85.88 |
| Total dry gas, wt% | 2.43 | 2.35 | 2.37 | 2.31 | 2.50 | 2.33 |
| Total LPG, wt% | 19.93 | 19.38 | 19.61 | 19.13 | 19.69 | 19.19 |
| LPG olefinicity, wt/wt | 0.68 | 0.69 | 0.69 | 0.69 | 0.68 | 0.70 |
| Total C4=, wt% | 7.66 | 7.51 | 7.73 | 7.51 | 7.52 | 7.62 |
| C3 olefinicity, wt/wt | 0.84 | 0.84 | 0.85 | 0.85 | 0.84 | 0.85 |
| C4 olefinicity, wt/wt | 0.60 | 0.60 | 0.61 | 0.61 | 0.59 | 0.61 |

^1^ Total valuable liquids defined as LPG + gasoline + LCO

Table S2. Catalytic cracking evaluation of all feeds at constant coke (2.5 wt%)

|  | Standard | MSW1 | MSW2 | MSW3 | BIO1 | MIX1 |
| --- | --- | --- | --- | --- | --- | --- |
| Process variables | | | | | | |
| Catalyst to oil ratio, wt/wt | 5.89 | 5.77 | 5.62 | 6.21 | 5.60 | 6.02 |
| Conversion, wt% | 73.13 | 73.66 | 73.24 | 74.86 | 73.34 | 74.24 |
| Hydrocarbon yields | | | | | | |
| H_2_, wt% | 0.08 | 0.08 | 0.07 | 0.08 | 0.07 | 0.08 |
| Methane, wt% | 0.82 | 0.75 | 0.78 | 0.78 | 0.79 | 0.78 |
| Ethane, wt% | 0.56 | 0.52 | 0.56 | 0.55 | 0.54 | 0.56 |
| Ethylene, wt% | 0.75 | 0.70 | 0.73 | 0.74 | 0.77 | 0.75 |
| Propane, wt% | 0.97 | 0.91 | 0.91 | 0.95 | 0.94 | 0.93 |
| Propylene, wt% | 5.56 | 5.29 | 5.46 | 5.46 | 5.39 | 5.43 |
| *n*-Butane, wt% | 0.81 | 0.76 | 0.76 | 0.80 | 0.79 | 0.78 |
| *i*-Butane, wt% | 3.77 | 3.57 | 3.55 | 3.73 | 3.65 | 3.62 |
| *n*-Butenes, wt% | 5.15 | 4.94 | 5.19 | 5.10 | 5.07 | 5.13 |
| *i*-Butylene, wt% | 2.24 | 2.19 | 2.34 | 2.24 | 2.23 | 2.29 |
| Gasoline, wt% | 49.91 | 51.44 | 50.41 | 51.93 | 50.59 | 51.40 |
| LCO, wt% | 15.98 | 15.76 | 16.06 | 15.28 | 15.93 | 15.67 |
| Bottoms, wt% | 10.88 | 10.57 | 10.70 | 9.86 | 10.73 | 10.09 |
| Coke, wt% | 2.50 | 2.50 | 2.50 | 2.50 | 2.50 | 2.50 |
| Calculated values |  |  |  |  |  |  |
| Total valuable liquids^1^, wt% | 84.40 | 84.87 | 84.67 | 85.49 | 84.59 | 85.25 |
| Total dry gas, wt% | 2.21 | 2.06 | 2.14 | 2.15 | 2.17 | 2.16 |
| Total LPG, wt% | 18.51 | 17.66 | 18.20 | 18.29 | 18.08 | 18.18 |
| LPG olefinicity, wt/wt | 0.70 | 0.70 | 0.71 | 0.70 | 0.70 | 0.71 |
| Total C4=, wt% | 7.39 | 7.13 | 7.53 | 7.34 | 7.31 | 7.41 |
| C3 olefinicity, wt/wt | 0.85 | 0.85 | 0.86 | 0.85 | 0.85 | 0.85 |
| C4 olefinicity, wt/wt | 0.62 | 0.62 | 0.64 | 0.62 | 0.62 | 0.63 |

^1^ Total valuable liquids defined as LPG + gasoline + LCO

Table S3. List of dominant compounds identified in pyrolysis oils using GC-MS.

| Retention time (min) | Compound | MSW1 | MSW2 | MSW3 | BIO1 | MIX1 |
| --- | --- | --- | --- | --- | --- | --- |
| 3.79 | Benzene | x |  | x |  | x |
| 4.36 | 1-Heptene | x |  | x |  | x |
| 4.53 | heptane |  |  |  |  | x |
| 6.91 | heptane, 4-methyl |  |  |  |  | x |
| 7.28 | Toluene | x |  | x | x | x |
| 8.03 | 1-octene |  |  | x |  | x |
| 8.04 | Cyclopropane, pentyl- | x |  |  |  |  |
| 8.29 | octane |  |  |  |  | x |
| 9.98 | 2,4-Dimethyl-1-heptene | x |  | x |  | x |
| 10.71 | Furfural |  |  |  | x |  |
| 11.1 | Ethylbenzene | x |  |  | x | x |
| 11.6 | Benzene 1,3-dimethyl |  |  |  | x |  |
| 11.63 | p-xylene |  |  |  |  | x |
| 12.13 | 1-Nonene | x |  |  |  | x |
| 12.37 | nonane |  |  |  |  | x |
| 12.48 | o-xylene |  |  |  | x |  |
| 12.53 | Styrene | x | x |  |  | x |
| 13.55 | Benzene, (1-methylethyl)- | x |  |  |  | x |
| 13.65 | 2-cyclopenen-1one, 2-methyl |  |  |  | x |  |
| 14.27 | Butyrolactone |  |  |  | x |  |
| 15.83 | Phenol |  |  |  | x |  |
| 15.91 | 1-Decene | x |  | x |  |  |
| 15.96 | α-Methylstyrene | x | x | x |  |  |
| 18.02 | 2-cyclopenen-1one, 2-hydroxy-3-methyl |  |  |  | x |  |
| 18.26 | Indene |  |  | x |  |  |
| 18.44 | Phenol, 2-methyl |  |  |  | x |  |
| 18.59 | 2-Cyclopenten-1one, 2,3-dimethyl |  |  |  | x |  |
| 18.74 | 1-Decene, 2,4-dimethyl- | x |  |  |  |  |
| 18.91 | ethanol, 2-(octyloxy) |  |  |  |  | x |
| 19.23 | p-cresol |  |  |  | x |  |
| 19.29 | Phenol, 2-methyl |  |  |  | x |  |
| 19.339 | 1-undecanol |  |  |  |  |  |
| 19.35 | 1-Undecene | x |  | x |  | x |
| 19.53 | Undecane | x |  | x |  | x |
| 19.87 | Phenol, 2-methoxy |  |  |  | x |  |
| 20.5 | Phenol, 3,5-dimethyl |  |  |  | x |  |
| 21.69 | Phenol, 3,4-dimethyl |  |  |  | x |  |
| 21.872 | Benzoic acid |  | x |  |  |  |
| 22.49 | 1-Dodecene | x |  | x |  |  |
| 22.49 | cyclododecane |  |  |  |  | x |
| 22.66 | Dodecane | x |  | x |  |  |
| 22.91 | Catechol |  |  |  | x |  |
| 23.07 | Naphthalene |  |  | x |  |  |
| 23.24 | Cresol |  |  |  | x |  |
| 24.34 | Phenol, 2-ethyl-4-methyl |  |  |  | x |  |
| 24.96 | 1,2 Benzenediol, 3-methyl |  |  |  | x |  |
| 25.43 | 1-Tridecene | x |  |  |  | x |
| 25.56 | 2-Isopropyl-5-methyl-1-heptanol | x | x | x |  |  |
| 25.69 | Phenol, 4-ethyl-2methoxy |  |  |  | x |  |
| 26.06 | Trichloroacetic acid, hexadecyl ester |  |  | x |  |  |
| 26.07 | 1-heptanol, 2,4,-diethyl |  |  |  |  | x |
| 27.94 | Phenol, 2,6-dimethoxy |  |  |  | x |  |
| 28.181 | 1-hexadecanol |  | x |  |  |  |
| 28.19 | 1-Tetradecanol | x |  | x |  |  |
| 28.19 | 1-tetradecene |  |  |  |  | x |
| 28.33 | Tetradecane |  |  | x |  |  |
| 28.45 | 4-Ethylcatechol |  |  |  | x |  |
| 30.53 | 1,2,4, trimethoxybenzene |  |  |  | x |  |
| 30.79 | n-Pentadecanol | x |  | x |  |  |
| 30.8 | 1-pentadecene |  |  |  |  | x |
| 30.91 | Pentadecane | x |  | x |  |  |
| 30.66 | Phenol, 2-methoxy-6-2(2-propenyl) |  |  |  | x |  |
| 32.39 | 5-tert-butylpyrogallol |  |  |  | x |  |
| 33.25 | 1-Hexadecanol | x |  |  |  |  |
| 33.27 | cetene |  |  |  |  | x |
| 35.41 | Benzene, 1,1'-(1,3-propanediyl)bis- | x | x |  |  | x |
| 35.59 | n-Heptadecanol-1 |  |  | x |  |  |
| 35.6 | 1-Nonadecene | x |  |  |  |  |
| 35.68 | Heptadecane |  |  | x |  |  |
| 35.8 | Benzene, 1-1'-(1-methyl-1,3-propanediyl)bis- |  | x |  |  |  |
| 36.8 | Phenol, 2-6-dimethoxy-4-(2-propenyl) |  |  |  | x |  |
| 37.404 | 1,2,-diphenylcyclopropane |  | x |  |  |  |
| 37.82 | heneicosylformate |  |  |  |  | x |
| 37.82 | 1-Octadecanol | x |  |  |  |  |
| 37.9 | Octadecane |  |  | x |  |  |
| 39.931 | 1-nonadecene |  | x | x |  |  |
| 40.319 | 1H-indene, 1-(phenylmethylene) |  | x |  |  |  |
| 40.79 | Hexadecnoic acid, methyl ester |  |  |  | x |  |
| 40.87 | Caffeine |  |  |  | x |  |
| 41.948 | 1-Octadecanol |  | x |  |  |  |
| 42.022 | Eicosane |  | x |  |  |  |
| 42.95 | Naphthalene, 2-phenyl- | x | x |  |  | x |
| 43.946 | Heneicosane |  | x |  |  |  |
| 44.22 | 13-Octadecenoic acid, methyl ester |  |  |  | x |  |
| 45.75 | Docosyl heptafluorobutyrate |  |  | x |  |  |
| 47.556 | Octacosane |  | x |  |  |  |
| 48.392 | Fumaric acid, 2,2-dichloroethyl pentadecyl ester |  | x |  |  |  |
| 49.259 | Tetracosane |  | x |  |  | x |
| 52.48 | Pentatriacontane |  |  | x |  |  |
| 52.475 | Pentacosane |  | x |  |  |  |
| 54.01 | Hentriacontane |  |  | x |  |  |
| 55.469 | Tetracontane |  | x |  |  |  |
| 56.91 | Tetratetracontane |  |  | x |  |  |
| 58.64 | 1,1':3',1''-Terphenyl, 5'-phenyl- | x |  |  |  |  |

Table S3. Scenarios 1 and 2 calculations using catalyst to oil ratio of 7 data

| Scenario 2: Applying standard conversion to unconverted pyrolysis oil fractions | | | | | | |
| --- | --- | --- | --- | --- | --- | --- |
|  | **Standard** | **MSW1** | **MSW2** | **MSW3** | **BIO1** | **MIX1** |
| Conversion | 75.8 | 76.2 | 75.9 | 76.1 | 76.3 | 76.2 |
| Unconverted products in pyoil (UPP)  Sum of diesel and bottoms fraction |  | 56 | 94 | 51 | 72 | 60 |
| Conversion of UPP  Standard conversion * UPP |  | 42.4 | 71.3 | 38.7 | 54.6 | 45.5 |
| Remaining UPP  UPP – conversion of UPP |  | 13.6 | 22.7 | 12.3 | 17.4 | 14.5 |
| Theoretical conversion  0.9 * standard conversion + 0.1 * (100 – remaining UPP) |  | 76.9 | 75.9 | 77.0 | 76.5 | 76.8 |
| Scenario 1: Assuming pure carryover of pyrolysis oil and no further conversion | | | | | | |
|  | **Standard** | **MSW1** | **MSW2** | **MSW3** | **BIO1** | **MIX1** |
| Conversion | 75.8 | 76.2 | 75.9 | 76.1 | 76.3 | 76.2 |
| Pyoil natural conversion  Naphtha content |  | 44 | 6 | 49 | 28 | 40 |
| Theoretical conversion  0.9 * standard conversion + 0.1 * pyoil natural conversion |  | 72.62 | 68.82 | 73.12 | 71.02 | 72.22 |
| Comparison of scenarios | | | | | | |
|  |  | **MSW1** | **MSW2** | **MSW3** | **BIO1** | **MIX1** |
| Scenario 1 |  | 72.62 | 68.82 | 73.12 | 71.02 | 72.22 |
| Scenario 2 |  | 76.9 | 75.9 | 77.0 | 76.5 | 76.8 |
| Experimental conversion |  | 76.2 | 75.9 | 76.1 | 76.3 | 76.2 |
